# Prolonged Response of a Patient with Relapsed Acute Myeloid Leukemia to a Novel Oral Bromodomain Extraterminal Inhibitor (BETi)

**DOI:** 10.1155/2020/8830123

**Published:** 2020-12-15

**Authors:** Meilen Chang Muñoz, Jennifer A. Murphy, Johannes E. Wolff, Brian A. Jonas

**Affiliations:** ^1^Department of Internal Medicine, University of California Davis, School of Medicine, Sacramento, CA, USA; ^2^Department of Pharmacy Services, University of California Davis Medical Center, Sacramento, CA, USA; ^3^University of California Davis Comprehensive Cancer Center, Sacramento, CA, USA; ^4^AbbVie Inc., North Chicago, IL, USA

## Abstract

Acute myeloid leukemia (AML) is an aggressive clonal bone marrow cancer characterized by high rates of relapse and mortality. A middle-aged woman with AML relapsed twice after achieving complete remission with induction therapy and subsequent salvage therapy. She was then enrolled in a clinical trial with the bromodomain extraterminal inhibitor (BETi) mivebresib and achieved complete remission with incomplete count recovery (CRi) with monotherapy. Subsequently, she relapsed and was transitioned to combination therapy with mivebresib plus venetoclax and achieved CR again. The patient required eltrombopag to decrease platelet dependence in both arms of the trial and exhibited less myelosuppression with the combination therapy. The exceptional response to mivebresib demonstrated by this patient underscores the therapeutic potential of mivebresib.

## 1. Introduction

Acute myeloid leukemia (AML) is an aggressive and heterogeneous bone marrow cancer driven by genetic and epigenetic alterations. It is the second most common type of adult leukemia making up approximately 30% of all cases and is the leading cause of leukemia-related deaths [1, 2]. Risk factors for development of AML include age >65, male gender, smoking, prior treatment with chemotherapy or radiation, certain environmental exposures, and history of antecedent myeloid disorder, such as myelodysplastic syndrome [3, 4]. The 5-year overall survival rate ranges from 40–50% in younger patients to 20–30% in older patients who receive high-intensity chemotherapy [5]. Relapse rates vary from 30–35% in patients aged <60 with favorable risk factors, up to 90% in patients aged >60 with adverse risk factors [6]. If the disease relapses, salvage therapy may be offered to achieve remission again, but survival in the relapsed setting is very poor [7]. Given the poor outcomes seen with AML, especially in the relapsed setting, it is imperative to find new strategies to improve remission and overall survival rates.

Bromodomain (BRD) and extraterminal (BET) proteins are a family of proteins that recognize acetylated lysine residues on histones to promote chromosome remodeling, histone modification or recognition and control transcription machinery to upregulate gene expression driving oncogenesis [8, 9]. Preclinical studies demonstrate that BET inhibitors (BETis) have antiproliferative activity and trigger a strong apoptotic response in cell lines derived from hematologic malignancies through the downregulation of MYC [10]. Thus, BET proteins have been identified as key targets for leukemia therapy [11, 12]. Mivebresib is a potent oral BETi that has recently entered phase I clinical trials [13]. It functions in a variety of mechanisms including G1 cell cycle arrest, induction of apoptosis, and potentially targeting the tumor microenvironment to provide additional therapeutic benefit. Another agent that has demonstrated high response rates and encouraging remission durations in AML is venetoclax, an oral B-cell lymphoma-2 (BCL-2) inhibitor that can activate apoptosis in malignancies [14]. Venetoclax is approved in combination with a hypomethylating agent (HMA) or low-dose cytarabine (LDAC) backbone in older patients with newly diagnosed AML or those who are not candidates for intensive chemotherapy [15, 16].

Like other systemic therapies, mivebresib and venetoclax cause myelosuppression that can lead to transfusion dependence. Allogeneic blood transfusions are associated with transfusion-related organ injury and sepsis, hemolytic transfusion reactions [17], platelet-refractory alloimmunization [18], and increased graft-versus-host disease after allogeneic hematopoietic stem cell transplantation [19] and hinder finding a suitable stem cell donor. Hence, bone marrow growth factors have been used to promote blood count recovery and decrease transfusion dependency. For example, eltrombopag is a potent thrombopoietin receptor agonist that has been studied for use in AML among other diseases characterized by thrombocytopenia [20].

In this study, we report the exceptional clinical response of a patient with multiply relapsed AML who was treated with mivebresib as monotherapy and subsequently after relapse with mivebresib in combination with venetoclax and eltrombopag. Complete remission with incomplete count recovery (CRi) was achieved with monotherapy, and after relapse she achieved a second CRi with combination therapy.

## 2. Case Description

A 60-year-old woman with no significant past medical history presented with *de novo* AML discovered on routine CBC. She presented with night sweats, bruising, and pancytopenia. The initial bone marrow biopsy revealed 66% myeloblasts, 30–40% cellularity, normal karyotype, and negative molecular studies. She achieved complete remission (CR) with 7 + 3 induction therapy and proceeded with 4 cycles of high-dose cytarabine consolidation. She remained in remission for nearly 20 months when a surveillance bone marrow biopsy revealed relapsed disease. She subsequently underwent salvage reinduction and achieved a second CR. The patient declined allogenic hematopoietic stem cell transplant in favor of consolidation therapy with intermediate-dose cytarabine. She remained in CR for approximately 10 months without additional therapy before relapsing for a second time.

**Figure 1:**
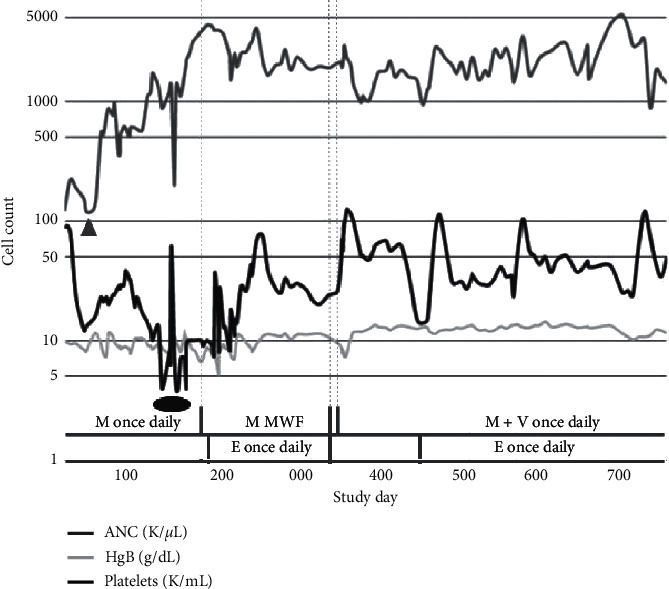
Absolute neutrophil count (ANC), hemoglobin (Hgb), and platelet counts throughout mivebresib mono- or combination therapy with venetoclax. ANC was less than 100/*μ*L during days 1 to 84, corresponding to cycles 1 to 3 of monotherapy (as denoted by grey triangle), and recovered to greater than 1,000/*μ*L, thereafter. Hgb generally remained between 7 and 12 g/dL throughout all treatment modalities. Platelets remained less than 10,000/*μ*L between days 140 and 190, corresponding to cycles 4 and 6 of monotherapy (as denoted by black oval), and then increased to 15,000 to 120,000/*μ*L for the duration of the trial. M = mivebresib; V = venetoclax; E = eltrombopag.

**Figure 2:**
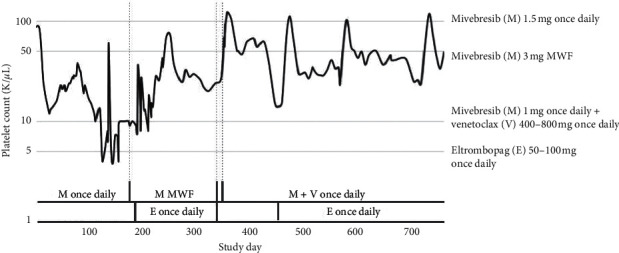
Platelet count response to therapy. Platelets decreased to less than 10,000/*μ*L with mivebresib 1.5 mg once daily monotherapy. Platelets increased to greater than 10,000/*μ*L with a change in mivebresib dosing to 3 mg MWF and increased further to greater than 50,000/*μ*L with the addition of eltrombopag. Platelet counts remained greater than 15,000/*μ*L and up to 120,000/*μ*L with the combination mivebresib and venetoclax and sustained with eltrombopag.

The patient subsequently elected to enroll in a phase I clinical trial of mivebresib for relapsed or refractory AML (NCT02391480) and was treated with mivebresib monotherapy. Her disease did not initially respond with an increase in marrow blasts from 21% to 40% after the first cycle, but this was followed by a decrease to 11% after cycle 2 and eventual CRi, due to ongoing thrombocytopenia, by the end of cycle 5. The patient first developed thrombocytopenia during cycle 2 and became transfusion dependent by cycle 5 (Figure 1 and Table 1). Eltrombopag 50 mg daily was added during cycle 7, and mivebresib was modified from 1.5 mg daily to 3 mg MWF during cycle 8 resulting in decreased transfusion requirements (Figure 2). Eltrombopag was increased to 100 mg daily, eliminating transfusion dependency, but the patient remained thrombocytopenic. Prior to the start of cycle 13 of mivebresib monotherapy, the patient relapsed with 14% blasts. She then discontinued mivebresib monotherapy and, after a washout period, was enrolled in a drug combination cohort of the study. The patient initially achieved CRi after cycle 1 of treatment with mivebresib and venetoclax (target dose 800 mg) before improving to CR after cycle 8 (Table 1). Eltrombopag was held during the early cycles of combination therapy as she maintained a platelet count greater than 50,000/*μ*L. However, during cycle 4 of mivebresib and venetoclax, the patient developed leukopenia and thrombocytopenia and eltrombopag was resumed. Platelet counts remained greater than 20,000/*μ*L and eventually improved to greater than 100,000/*μ*L with eltrombopag, mivebresib, and venetoclax. In contrast, platelet counts generally had previously remained below 30,000/*μ*L with eltrombopag and mivebresib monotherapy.

Pretrial genomic screening revealed a STAG2 R213Efster12 mutation with 18% variant allele frequency (VAF) (Table 1). Genomics performed during cycle 11 of monotherapy revealed persistence of STAG2 mutation with a decrease in VAF to 6.6% and a new mutation WT1 K467Ter (VAF 7.4%). Genomics performed during cycle 5 of combination therapy did not detect any mutations. However, a KMT2A I3432V (VAF 50.6%) variant of unknown significance was detected after cycle 13, and the patient relapsed soon thereafter.

## 3. Discussion

AML comprises ∼30% of all adult leukemia cases, and relapse rates can be as high 90% in elderly patients with adverse risk factors. High relapse rates necessitate novel systemic therapies with longer remission durations. New targets have been identified that can induce apoptosis in malignant cells, including BET and BCL-2 proteins. Mivebresib is a pan-inhibitor of the BET proteins that is currently in phase I clinical trials. In this case, mivebresib was initially used as a monotherapy per protocol with the patient achieving CRi after completion of cycle 5. Given that the patient continued to experience severe thrombocytopenia and transfusion dependence, which prevented the patient from achieving CR, as defined by <5% blasts by morphology in a cellular marrow with recovery of both neutrophils and platelets, eltrombopag was added as a supportive measure. This resulted in decreased transfusion dependency, but the thrombocytopenia persisted. At disease relapse, mivebresib monotherapy was discontinued, and after a washout period, combination therapy with venetoclax and mivebresib was started. The patient once again achieved CRi during cycle 2 before improving to a CR after cycle 8. Response was maintained for approximately 11 months until cycle 13 when there was morphologic relapse.

Interestingly, platelet counts were not as low with combination therapy as with monotherapy, and the patient sustained only mild to moderate thrombocytopenia with the addition of eltrombopag to venetoclax and mivebresib. She did not require any platelet transfusions with this combination regimen. Possible explanations for this observation include a deeper response to the combination therapy resulting in improved count recovery, response related to the venetoclax component alone, or that combination therapy may have resulted in a more effective response to eltrombopag potentially attributable to a synergistic effect between the three drugs.

Studies demonstrate that eltrombopag stimulates thrombopoiesis via the JAK-STAT signaling cascade [21]. This same pathway regulates expression of the apoptosis regulatory genes BCL-2 [22] and MYC [23]. Given that venetoclax is a BCL-2 inhibitor and mivebresib downregulates MYC expression, we can hypothesize that venetoclax and mivebresib downregulate the apoptotic pathway in platelets, allowing the cell proliferation pathway to predominate. Additional studies are required to explore these potential mechanisms of response and synergy.

In addition, we can consider the contribution of STAG2 deficiency on incomplete count recovery. STAG2 is a part of the cohesin multimeric protein complex responsible for cohesion of sister chromatids, transcription regulation, and homologous DNA repair [24]. Preliminary data suggest that loss of STAG2 can lead to dysregulated transcription in AML [25]. Considering that our patient initially expressed a STAG2 mutation, it is conceivable that STAG2 deficiency globally prevented appropriate transcription, inevitably inhibiting effective platelet production. Once this mutation resolved in the setting of combination therapy, we posit that eltrombopag-induced platelet production was more efficient. Given resolution of this mutation with combination therapy, we can also consider the potential for STAG2 to be a predictive marker for response to mivebresib-based therapy.

## 4. Conclusion

In this case study of an exceptional responder, mivebresib as monotherapy and in combination with venetoclax conferred an 18-month survival supporting the potential clinical activity of this novel BETi in relapsed or refractory AML. In combination with mivebresib and/or venetoclax, eltrombopag also led to decreased transfusion dependence, which can augment quality of life, decrease healthcare costs, and reduce platelet alloimmunization. Primary data analyses of all patients in the AML cohorts investigated in this trial, including safety and clinical efficacy, will be described elsewhere.

## Figures and Tables

**Table 1:** Clinical course, including bone marrow, molecular, and peripheral blood responses.

Day	Circulating blasts (%)	BM blast morphology (%)	MLFS achieved	MRD flow blasts (%)	Cytogenetics	Molecular	ANC (K/*μ*L)	Hgb (g/dL)	Plt (K/*μ*L)	ELN response
Screening mono (M)	6	21	N/A	Not done	46,XX [20]	STAG2 R213Efster12 (VAF 18%)	0.12	10.6	95	N/A
C2D1M	3	40	No	Not done	46,XX [18]	Not done	0.12	8	13	Resistant disease
C3D1M	0	11	No	Not done	Failed	Not done	0.76	7.5	23	Resistant disease
C5D1M	0	8	No	Not done	46,XX [20]	Not done	1.2	9	12	Resistant disease
C6D1M	0	4	Yes	20.1	46,XX [20]	Not done	1.34	8.9	4	CRi
C7D1M	0	4	Yes	10.8	46,XX [20]	Not done	3.3	8.2	10	CRi
C9D1M	0	8	No	12.1	46,XX [20]	Not done	2.5	10.9	28	Morphologic relapse
C10D1M	0	4	Yes	Not done	46,XX [20]	Not done	3.1	8.6	43	CRi
C11D1M	0	8	No	13.5	46,XX [20]	STAG2 R213Efster12 (VAF 6.6%), WT1 K467Ter (VAF 7.4%)	2.7	11.4	33	Morphologic relapse
C13D1M	0	14	No	26.3	46,XX [20]	Not done	1.9	10.6	24	Resistant disease
Screening combo (C)	Not reported	8	N/A	Not done	46,XX [20]	Not done	>1.0	9.3	29	N/A
C2D1C	0	2	Yes	0.99	46,XX [20]	Not done	1.1	11.9	51	CRi
C3D1C	0	2	Yes	0.64	46,XX [20]	Not done	1.8	12.8	68	CRi
C5D1C	0	1	Yes	0.35	46,XX [20]	None detected	1.3	12	20	CRi
C7D1C	0	2	Yes	0.14	46,XX [20]	Not done	1.5	12.2	30	CRi
C9D1C	0	1	Yes	0.34	46,XX [20]	Not done	3.5	12.9	104	CR
C11D1C	0	N/A	N/A	0.9	46,XX [20]	None detected	3	13.7	51	CRi
C13D1C	0	6	No	5.3	46,XX [20]	Not done	4.4	13.3	42	Morphologic relapse
C14D1C	0	4	Yes	5.5	46,XX [20]	KMT2A I3423V (VUS, VAF 50.6%)	2.9	10.2	24	CRi
C15D1C	0	10	No	10.7	46,XX [20]	Not done	1.4	11.5	50	Morphologic relapse
EOT marrow deferred							2.6	11.7	75	

BM, bone marrow; MLFS, morphologic leukemia-free state; MRD, measurable residual disease; VAF, variant allele frequency; ANC, absolute neutrophil count; Hgb, hemoglobin; Plt, platelet; ELN, European LeukemiaNet; CRi, complete remission with incomplete count recovery; CR, complete remission; N/A, not applicable; EOT, end of treatment; VUS, variant of uncertain significance.

## Data Availability

The data supporting the conclusions for this study are confidential and protected by under HIPAA Privacy Rule compliance measures.
